# A necroptosis-related prognostic model for predicting prognosis, immune landscape, and drug sensitivity in hepatocellular carcinoma based on single-cell sequencing analysis and weighted co-expression network

**DOI:** 10.3389/fgene.2022.984297

**Published:** 2022-09-21

**Authors:** Jingjing Li, Zhi Wu, Shuchen Wang, Chan Li, Xuhui Zhuang, Yuewen He, Jianmei Xu, Meiyi Su, Yong Wang, Wuhua Ma, Dehui Fan, Ting Yue

**Affiliations:** ^1^ Department of Anesthesiology, Jincheng People’s Hospital, Jincheng, Shanxi, China; ^2^ Department of Anesthesiology, The First Affiliated Hospital of Guangzhou University of Chinese Medicine, Guangzhou, China; ^3^ Department of General Surgery, Jincheng People’s Hospital, Jincheng, Shanxi, China; ^4^ The Fifth Clinical Medical School, Guangzhou University of Chinese Medicine, Guangzhou, China; ^5^ Department of Rehabilitation, GuangDong Second Traditional Chinese Medicine Hospital, Guangzhou, China; ^6^ Department of Oncology Rehabilitation, Jincheng People’s Hospital, Jincheng, Shanxi, China

**Keywords:** prognostic model, hepatocellular carcinoma, necroptosis, therapy, nomogram

## Abstract

**Background:** Hepatocellular carcinoma (HCC) is a highly lethal cancer and is the second leading cause of cancer-related deaths worldwide. Unlike apoptosis, necroptosis (NCPS) triggers an immune response by releasing damage-related molecular factors. However, the clinical prognostic features of necroptosis-associated genes in HCC are still not fully explored.

**Methods:** We analyzed the single-cell datasets GSE125449 and GSE151530 from the GEO database and performed weighted co-expression network analysis on the TCGA data to identify the necroptosis genes. A prognostic model was built using COX and Lasso regression. In addition, we performed an analysis of survival, immunity microenvironment, and mutation. Furthermore, the hub genes and pathways associated with HCC were localized within the single-cell atlas.

**Results:** Patients with HCC in the TCGA and ICGC cohorts were classified using a necroptosis-related model with significant differences in survival times between high- and low-NCPS groups (*p* < 0.05). High-NCPS patients expressed more immune checkpoint-related genes, suggesting immunotherapy and some chemotherapies might prove beneficial to them. In addition, a single-cell sequencing approach was conducted to investigate the expression of hub genes and associated signaling pathways in different cell types.

**Conclusion:** Through the analysis of single-cell and bulk multi-omics sequencing data, we constructed a prognostic model related to necroptosis and explored the relationship between high- and low-NCPS groups and immune cell infiltration in HCC. This provides a new reference for further understanding the role of necroptosis in HCC.

## Introduction

Primary liver cancer is the sixth most common cancer in the world and the second leading cause of cancer-related death (Yang et al., 2019). Hepatocellular carcinoma (HCC) is the most common type of primary liver cancer (Chaudhary et al., 2019). Most HCC patients are diagnosed at an advanced stage. The gold standard treatments, including tumor resection, local ablation with radiofrequency, and sometimes liver transplantation, have low success rates with high relapse rates and short survival times (Dhanasekaran et al., 2016). Additionally, patients with HCC who present with similar tumor, lymph node, and metastasis (TNM) stage have different clinical outcomes, and there are few current effective prognostic indicators.

Recent research has demonstrated the importance of the tumor microenvironment (TME) in promoting tumor aggressiveness (Altorki et al., 2019). The survival of patients with various malignancies can be prolonged by immune checkpoint inhibitors. However, many patients with HCC currently often respond poorly to immune checkpoint inhibitors, which may be due to low mutational loads, acquiring new immune checkpoints, and producing immunosuppressive factors (Riley et al., 2019). Therefore, there is a need to identify new biomarkers for HCC as well as to comprehend their significance in TME.

Programmed cell death has a strong impact on the characterization of the TME ecosystem (Chevrier et al., 2017). Resistance to apoptosis, a problem affecting cancer development, is one of the hallmarks of cancer (Reyna et al., 2017). In the process of cancer cell resistance to death, growth signals are overactivated, the metabolism is reprogrammed, and a change in the immune microenvironment occurs (Sahin et al., 2017). Inducing cancer cell death is becoming increasingly popular as a potential cancer treatment method (Bersuker et al., 2019). HCC cells can die by several different mechanisms, including apoptosis and necroptosis (NCPS) (Yuan et al., 2019). Both mechanisms play a significant role in homeostasis, inflammation, anti-infection, and tumorigenesis (Karki et al., 2021; Koren and Fuchs, 2021).

Necroptosis was once believed to be the “accidental death” of cells. However, current research indicates that necroptosis is distinct from conventional apoptosis (González-Juarbe et al., 2017). Necroptosis leads to membrane destabilization, which subsequently precedes swelling and lysis of cells, resulting in the release of intracellular constituents (González-Juarbe et al., 2017). Inhibited caspase 8 and receptor-interacting serine/threonine protein kinase 1 (RIPK1) are both involved in necroptosis pathway activation via recruitment and activation of receptor-interacting serine/threonine protein kinase 3 (RIPK3) (Alvarez-Diaz et al., 2016). Necroptosis occurs when caspase 8 is inactivated or absent, resulting in the activation and autophosphorylation of RIPK1 and RIPK3(Tanzer et al., 2017). During this process, the cell membrane ruptures, and the contents are released, stimulating an immune response (Kalliolias and Ivashkiv, 2016). Necroptosis becomes attractive as an alternative to apoptosis for killing tumor cells if apoptosis fails to kill them (Kalliolias and Ivashkiv, 2016). As well, the immune microenvironment is positively impacted by necroptosis (Gong et al., 2019).

Interestingly, the role of necroptosis in cancer is complex. In general, high levels of necroptosis result in strong adaptive immune responses that inhibit the progression of tumors. The recruitment of strong immune responses may also contribute to tumor progression (Koo et al., 2015; Najafov et al., 2017). Moreover, the inflammatory response may contribute to tumorigenesis and metastasis, as well as generate an immunosuppressive tumor microenvironment. Guo et al. (2022) has shown that loss of key necroptosis gene significantly reduces clinical symptoms of liver injury and fibrosis. Necroptosis has completely opposite effects on different types of cancer, the mechanism of which is still unclear. With the emergence of immune checkpoint therapy, changes in the immune microenvironment resulting from necroptosis are also important to consider. There is therefore a need to investigate the relationship between necroptosis and HCC.

Here, we downloaded the data of HCC patients from the TCGA and ICGC databases, as well as two single-cell datasets, GSE125449 (Ma et al., 2019) and GSE151530 (Ma et al., 2021), and one microarray dataset, GSE76427 (Grinchuk et al., 2018) from the GEO database. The TCGA cohort was used for model building. The ICGC cohort and GSE76427 were used to validate the results of our analysis. Two single-cell sequencing datasets, GSE125449 and GSE151530, were chosen for single-cell analysis because of their relatively large sample size and inclusion of clinical data. Through comprehensive bioinformatic analysis, we developed a prognostic model based on necroptosis and classified HCC patients into low- and high-risk groups, the results of which were significantly different. Furthermore, we explored the potential value of the signature in guiding the tumor mutational load, immune microenvironment, and drug sensitivity.

## Methods

### Download and processing of transcriptome data

**FIGURE 1:**
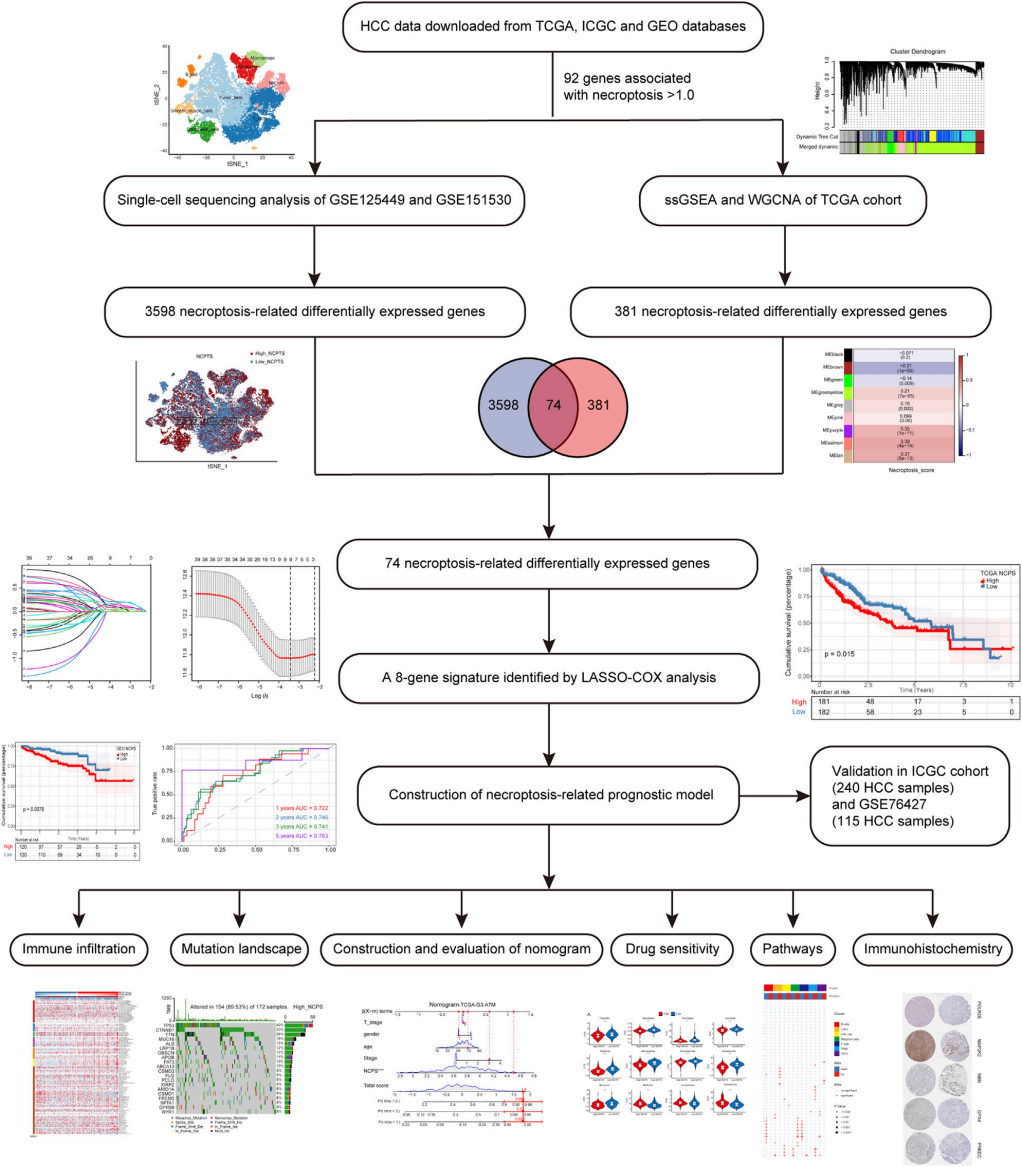
Flowchart of the analysis.

This flowchart illustrates the key steps in the analysis (Figure 1). The data of HCC were downloaded from TCGA (https://portal.gdc.cancer.gov/) as a training cohort (Grossman et al., 2016). Count data and TPM data of HCC were extracted using R software (4.2.0), and a total of 363 tumor samples with complete clinical data were obtained. The HCC dataset was downloaded through ICGC (https://dcc.icgc.org/) database as a validation cohort, and the count data type and TPM data type of HCC were extracted, and a total of 240 tumor samples were obtained with complete clinical information (Zhang et al., 2019). GSE76427, measured using the Illumina HumanHT-12 V4.0 expression beadchip, contained 115 HCC samples (Grinchuk et al., 2018). The raw CEL files for GSE76427 were downloaded from the GEO database. More details of the data processing are in Supplementary Material S1, S2.

### Download and processing of single-cell data

The single-cell datasets GSE125449 and GSE151530 for HCC were downloaded from the GEO database (https://www.ncbi.nlm.nih.gov/geo/) (Barrett et al., 2013). The GSE125449 dataset contains nine HCC samples and the GSE151530 dataset contains 32 HCC samples. We performed quality control on the data of all samples. We retained cells with genes expressed in at least 10 cells, less than 10% of mitochondrial genes, more than 200 genes, less than 5% hemoglobin genes, less than 50% ribosomal genes, and expression between 200 and 7000. We set a limit of 3000 highly variable genes. Next, we normalized all samples, removed batch effects, and integrated them by SCT. Then, using the tSNE method with the “DIMS” parameter set to 20, the dimensionality of the data was reduced. Cell clustering was then carried out using the “KNN” method with a resolution of 2.0. Subsequently, the cells were annotated with the Human Primary Cell Atlas (HPCA) from the “SingleR” package as a reference dataset (Mabbott et al., 2013). Finally, the proportion of NCPS-related genes in each cell can be calculated using the “PercateFeatureSet” function.

### Identification of NCPS-related genes

In the GeneCards database (https://www.genecards.org/), 614 genes associated with necroptosis were identified (Safran et al., 2021). A total of 92 genes were identified that had an association score of greater than 1.0 with necroptosis (Supplementary Material S3). Then, the NCPS-related genes were scored for each sample by the combined analysis of ssGSEA (Single Sample Gene Set Enrichment Analysis) and WGCNA (Weighted Co-Expression Network Analysis). The log2 processed data were used for ssGSEA analysis.

### ssGSEA

Gene sets enriched in a sample are often quantified by using the ssGSEA method with “GSVA” package (version: 1.44.2) (Bindea et al., 2013; Hänzelmann et al., 2013). In this study, ssGSEA analysis was utilized to determine the NCPS-related scores of each patient with HCC.

### WGCNA

WGCNA analysis is one method used in systems biology for determining patterns of genetic association among diverse samples (Langfelder and Horvath, 2008). In addition to identifying highly covariant genomes, WGCNA analysis can be used to identify potential biomarkers or therapeutic targets based on the correlation between genomes and phenotypes. In this study, gene modules associated with NCPS scores in HCC were found by “WGCNA” package (version: 1.71), and genes associated with necroptosis were obtained. Non-gray modules were identified by setting a soft threshold of eight, a minimum number of module genes of 80, and combining modules that had similarities of less than 0.3.

### Construction of NCPS-related prognostic model

First, univariate COX analysis was used to identify NCPS-related genes with prognostic values by using the “survival” package (version: 3.3-1). Next, a prognostic model was developed based on the least absolute shrinkage and selection operator (LASSO) regression for NCPS-related genes by using the “glmnet” package (version: 4.1-4) (Lossos et al., 2004; Friedman et al., 2010). In this way, the NCPS score could be calculated for each HCC sample by the formula. Gene expression levels were weighted by their respective coefficients of LASSO regression to calculate the NCPS score. The formula was as follows:

$$NCPS\;score=\overset{n}{\underset{i=1}{\sum }}C{oef}_{i}\times Exp_{i}$$ (1)

where *n*, *Exp*_*i*_, *Coef*_*i*_, represented the number, the expression value, and the coefficient of each selected gene, respectively. According to the median value of the TCGA-HCC cohort, patients could be classified into low- and high-risk groups. Thereafter, we assessed the accuracy of the model by comparing prognostic differences between the two groups.

### Validation of NCPS-related prognostic model

The ICGC cohort and GSE76427 were selected as the external validation cohorts. According to the formula of the prognostic model, NCPS scores for each sample were calculated, and patients were categorized based on their median NCPS scores into high-risk and low-risk groups. We then conducted a survival analysis comparing the high- and low-NCPS groups. Receiver operating characteristic (ROC) curves were utilized to evaluate the model’s accuracy by using the “timeROC” package (version: 0.4) (Li et al., 2018). To determine whether the model grouped HCC patients more effectively, principal component analysis (PCA) was performed using the “PCAtools” package (version: 2.8.0) and “scatterplot3d” package (version: 0.3-41).

### Immune infiltration and mutation landscape

We performed immune infiltration analysis of HCC patients in the TCGA database using immune cell infiltration algorithms from the IOBR package (version: 0.99.9) (Zeng et al., 2021). Next, we examined the differences in the levels of immune cell infiltration between the two NCPS groups and presented the immune cells with different levels of infiltration as a heat map. Also, the expression of immune checkpoint-related genes in the various NCPS subgroups was visualized by a boxplot. We identified the top 20 genes with the highest mutation rates by comparing the mutation rates between groups with high and low NCPS scores.

### Nomogram

Using clinical data and NCPS values, a nomogram was developed in this study to assess the probability of mortality in patients with HCC using the “rms” package (version: 6.3-0) and “regplot” package (version: 1.1). This nomogram was evaluated by using prognostic ROC curves and decision curve analysis (DCA) to determine its accuracy in predicting patient outcomes. The DCA analysis was performed using the “ggDCA” package (version: 1.1) (Vickers and Elkin, 2006).

### Drug sensitivity, immunohistochemistry, pathways

To improve personalized treatment, we calculated half maximal inhibitory concentrations (IC50) using the “pRRophetic” package (version: 0.5) and compared these data between high-risk and low-risk groups (Geeleher et al., 2014). Low IC_50_ values indicate greater drug effectiveness. The Human Protein Atlas (HPA) database (version: 21.1, http://www.proteinatlas.org/) is the most comprehensive database for assessing protein distribution in human tissues (Uhlén et al., 2015).

HPA database was used to obtain prognostic gene expression data. Immunohistochemical staining images of normal and HCC tissues were used to analyze the protein expression of genes. In addition, we performed an enrichment analysis of pathways associated with different cell types in the single-cell data and then mapped the significantly different pathways to tSNE plots for visualization. Pathway enrichment analysis was performed using the “irGSEA” package (version: 1.1.2). Finally, the pathways associated with HCC in the TCGA dataset were analyzed.

### Statistical analysis

Statistical analysis was performed using the R software (version 4.2.0). Continuous data were analyzed using Mann-Whitney tests, and categorical data were analyzed using Fisher’s exact tests. Pearson correlation coefficient was used to estimate the correlation between continuous variables. The Kaplan-Meier method was used for survival analysis. The Log-rank test was used to determine the significance of differences. All statistical analyses were considered significant if the *p*-values were less than 0.05.

## Results

### Annotation of single-cell sequencing data and identification of differentially expressed genes associated with NCPS

**FIGURE 2:**
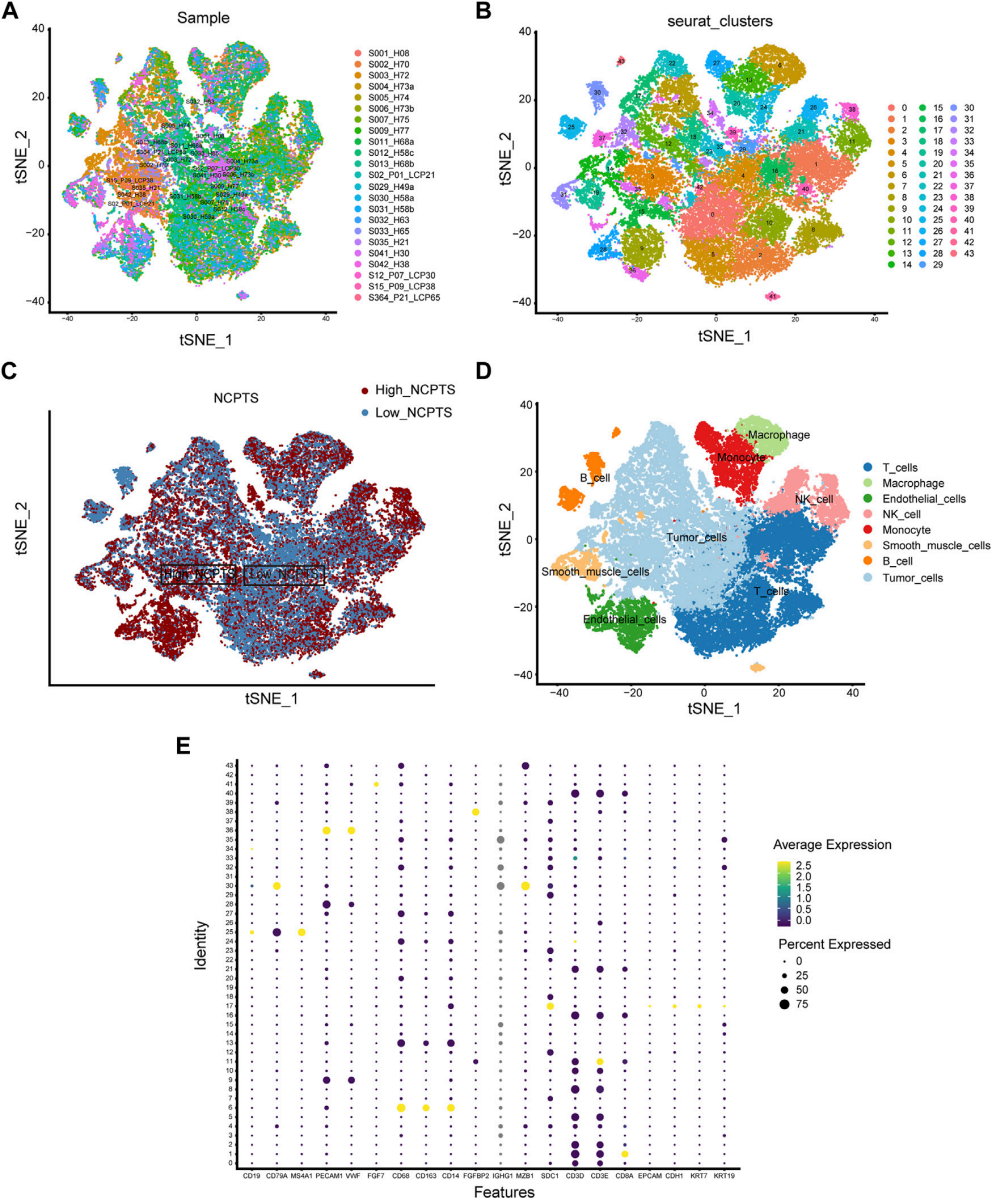
Single-cell analysis. **(A)** There were no significant batch effects in the 23 samples. **(B)** Dimensionality reduction and cluster analysis. **(C)** Percentage of genes in each cell that are involved in necroptosis. **(D)** Annotation of cells in accordance with their surface marker genes. **(E)** A list of the surface markers of the eight types of cells.

**FIGURE 3:**
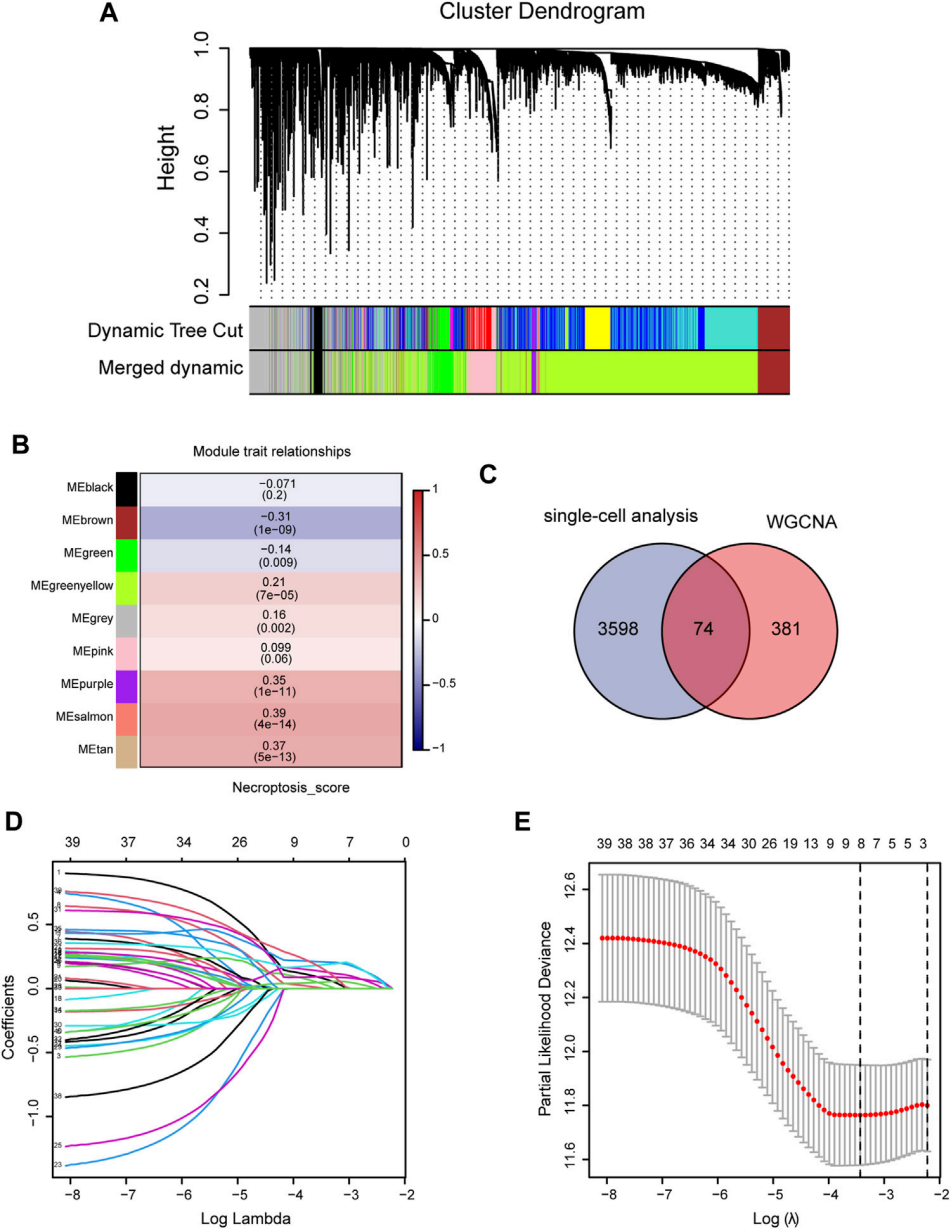
Construction of prognostic model. **(A,B)** WGCNA screening for modules relating to necroptosis. **(C)** The intersection between differential genes identified by single-cell analysis and genes identified by WGCNA. **(D,E)** Using Lasso regression, the final genes were selected for the prognostic model.

We first analyzed the single-cell sequencing datasets GSE125449 and GSE151530 for HCC to integrate different samples. As shown in Figure 2A, there were no significant batch effects in the 23 samples, and further analysis of the results could be conducted. The K-Nearest Neighbor (KNN) algorithm was used to divide all cells into 43 clusters (Figure 2B). After entering 92 genes related to NCPS using the “PercateFeatureSet” function, a percentage of the genes associated with NCPS was calculated for each cell. Cells were classified based on the median percentage of NCPS genes and represented as tSNE plots (Figure 2C). We then identified eight distinct cell types based on the expression of surface markers of different cell types in different clusters. They were T cells, macrophages, endothelial cells, NK cells, monocytes, smooth muscle cells, B cells, and tumor cells (Figure 2D). The surface markers for eight types of cells are shown in Figure 2E. Furthermore, we identified 3598 genes that were differentially expressed between high- and low-NCPS groups (Supplementary Material S4). Using the WGCNA analysis of 363 samples from the TCGA cohort, we have obtained gene modules associated with necroptosis. In total, eight non-gray modules were identified by setting a soft threshold of 8 (Figure 3A). As shown in Figure 3B, MEsalmon, MEtan, and MEpurple were strongly associated with the NCPS score. Further analysis was performed on the genes in these three modules.

### The NCPS-related prognostic model could be used to classify HCC patients and predict their prognosis

An intersection was drawn between differential genes derived from single-cell analysis and genes identified by WGCNA. In Figure 3C, 74 genes are shown as candidates for the next step in the analysis (Supplementary Material S5). Based on univariate COX analysis within the TCGA cohort, 45 genes have been identified as significantly associated with prognosis. The LASSO regression analysis employed a random seed of 2022, and the results indicated that gene contraction stabilized with minimal partial likelihood deviation when the number of genes included was 8 (Figures 3D,E). Table 1 summarizes the results of the Lasso regression for each of these genes. The prognostic model was constructed from eight genes, including RAD21, NBN, PRKDC, MAP2K2, RIPK2, BOP1, POLR2E, and GPX4. As follows was the prognostic model.

NCPS=RAD21*0.07523793+NBN*0.02974632+PRKDC*0.2080255+MAP2K2*0.13063826+RIPK2*0.05091183+BOP1*0.0940779+POLR2E*0.18787475+GPX4* 0.1444125

**TABLE 1:** Eight genes were identified by lasso regression to construct a prognostic model.

ID	Coef	Hazard_ratio	Low_CI	High_CI	*p*_value
RAD21	0.07523793	1.61760529	1.18401058	2.20998607	0.00252028
NBN	0.02974632	1.69458991	1.18308239	2.42724849	0.00401446
PRKDC	0.2080255	1.68776122	1.27783985	2.22918228	0.00022688
MAP2K2	0.13063826	1.83095476	1.27010213	2.6394691	0.00119002
RIPK2	0.05091183	1.61290563	1.22724908	2.11975272	0.00060646
BOP1	0.0940779	1.48888676	1.1924913	1.85895175	0.00044089
POLR2E	0.18787475	2.33187249	1.40355261	3.87418988	0.00108012
GPX4	0.1444125	1.69395714	1.12709302	2.54592189	0.01122826

**FIGURE 4:**
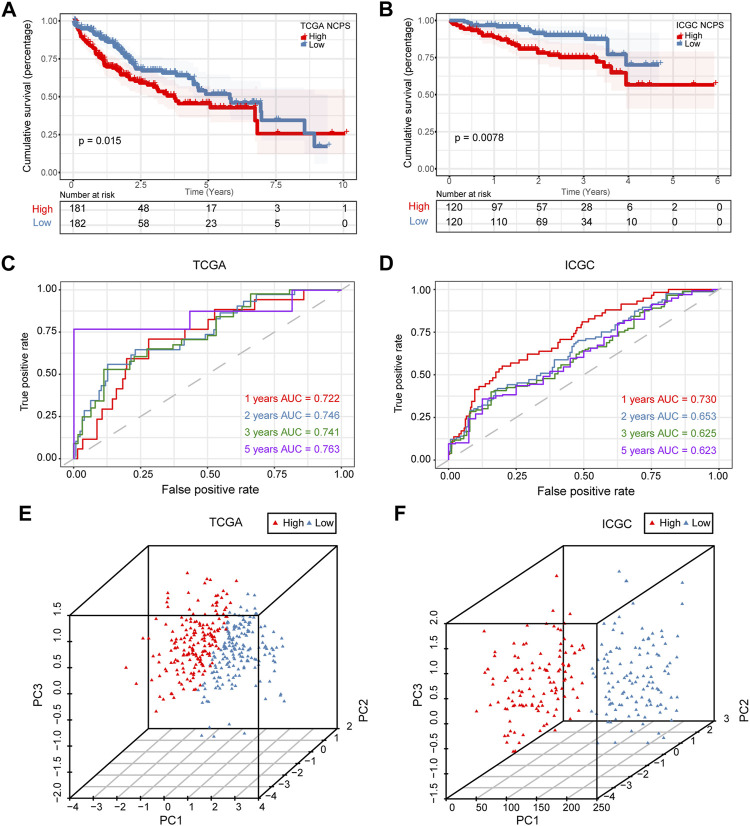
Validation of prognostic model. **(A)** Survival analysis of the training set showed significantly poorer outcomes for NCPS high group (*p* = 0.015). **(B)** Survival analysis results in the validation set were similar to those in the training set (*p* = 0.0078). **(C)** ROC curve of the training set. **(D)** ROC curve of the validation set. **(E,F)** 3D-PCA analysis in the training set and validation set.

Based on median values, patients were divided into high- and low-risk groups. Figure 4A showed that the high-NCPS group in the training cohort had a worse prognosis (*p* = 0.015). Figure 4B demonstrated that patients with high-NCPS had worse outcomes than those with low-NCPS in the validation cohort (*p* = 0.0078). ROC curves were generated for both the training and validation cohorts to test the prognosis assessment ability. As shown in Figure 4C, the area under the curve (AUC) values were 0.722, 0.746, 0.741, and 0.763 at 1, 2, 3, and 5 years in the TCGA cohort, respectively. In the validation cohort, AUC values were 0.730, 0.653, 0.625 and 0.623 at 1, 2, 3 and 5 years, respectively (Figure 4D). In the GSE76427 cohort, the results showed that the high-NCPS group had a worse prognosis (*p* = 0.0037), and AUC values were 0.682, 0.696, and 0.778 at 2, 3 and 5 years, respectively (Supplementary Material S6).

Based on these results, the NCPS-related prognostic model was found to be accurate in predicting the outcomes of patients in all three cohorts. Furthermore, PCA was performed on the eight genes included in all three cohorts, and the results were similar. The results showed that the model performed well in classifying HCC patients (Figures 4E,F).

### The nomogram could be more reliable in predicting patient outcomes than other indicators

**FIGURE 5:**
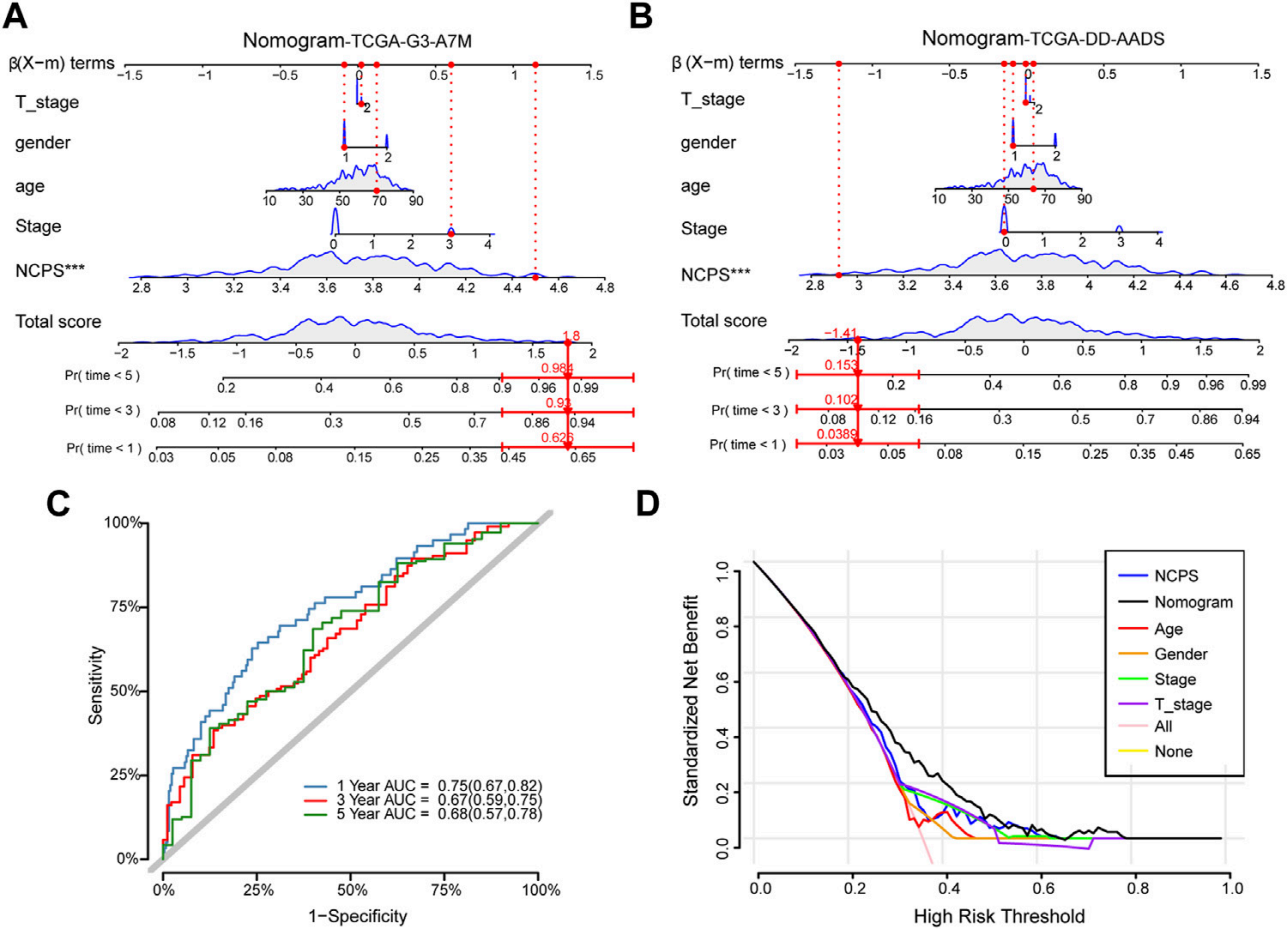
Construction of the nomogram. **(A)** High-NCPS patient “TCGA-G3-A7M9”: Mortality rates were estimated to be 0.626, 0.92, and 0.984 at 1, 3, and 5 years, respectively. **(B)** Low-NCPS patient “TCGA-DD-AADS”: Mortality rates in 1, 3, and 5 years were estimated to be 0.0389, 0.102, and 0.153, respectively. **(C)** The ROC curve for the nomogram. **(D)** DCA analysis showed that the nomogram was more effective than other clinical indicators.

Combining clinical information and NCPS scores, we constructed a nomogram that allows us to assess patients’ prognoses. In Figure 5A, the estimated mortality rates for patients with the high-NCPS score “TCGA-G3-A7M9” were 0.626, 0.92, and 0.984 at 1, 3, and 5 years based on gender, age, T-stage, and total stage (Table 2). Based on the low-NCPS score, the estimated mortality rates for patients with “TCGA-DD-AADS” were 0.0389, 0.102, and 0.153 at 1, 3, and 5 years based on sex, age, T-stage, and total stage (Figure 5B). In Supplementary Material S7, NCPS scores and clinical characteristics of 363 patients from the TCGA-HCC dataset are presented. Accordingly, a clinical decision could be based on assessing a patient’s risk and guiding their subsequent treatment. Furthermore, the accuracy of the nomogram was assessed through ROC analysis, which showed AUCs of 0.75, 0.67, and 0.68 for 1, 3, and 5 years, respectively (Figure 5C). In addition, we assessed the utility of the model to support clinical decision-making by using decision curve analysis (DCA) and reported the net clinical benefit of the model. The results showed that the nomogram is better than other clinical indicators, indicating that the nomogram is effective in predicting the patient’s prognosis (Figure 5D).

**TABLE 2:** Comparison of clinical data from patients with high- and low-NCPS in the TCGA-HCC dataset.

Patient ID	TCGA-G3-A7M9	TCGA-DD-AADS
NCPS score	4.501	2.920
Gender	Male	male
Status	Dead	Alive
Age (year)	70.104	63.636
M_stage	MX	M0
N_stage	NX	N0
Stage	Stage IIIB	Stage I
T_stage	T3b	T1
Survival time (year)	0.153	1.299
1-year mortality rates	CI: 0.626 (0.432, 0.82)	CI: 0.0389 (0.0234, 0.0646)
3-year mortality rates	CI: 0.93 (0.782, 0.99)	CI: 0.102 (0.0617, 0.165)
5-year mortality rates	CI: 0.984 (0.906, 0.999)	CI: 0.153 (0.094, 0.243)

### Survival analysis and cellular localization of the eight hub genes

**FIGURE 6:**
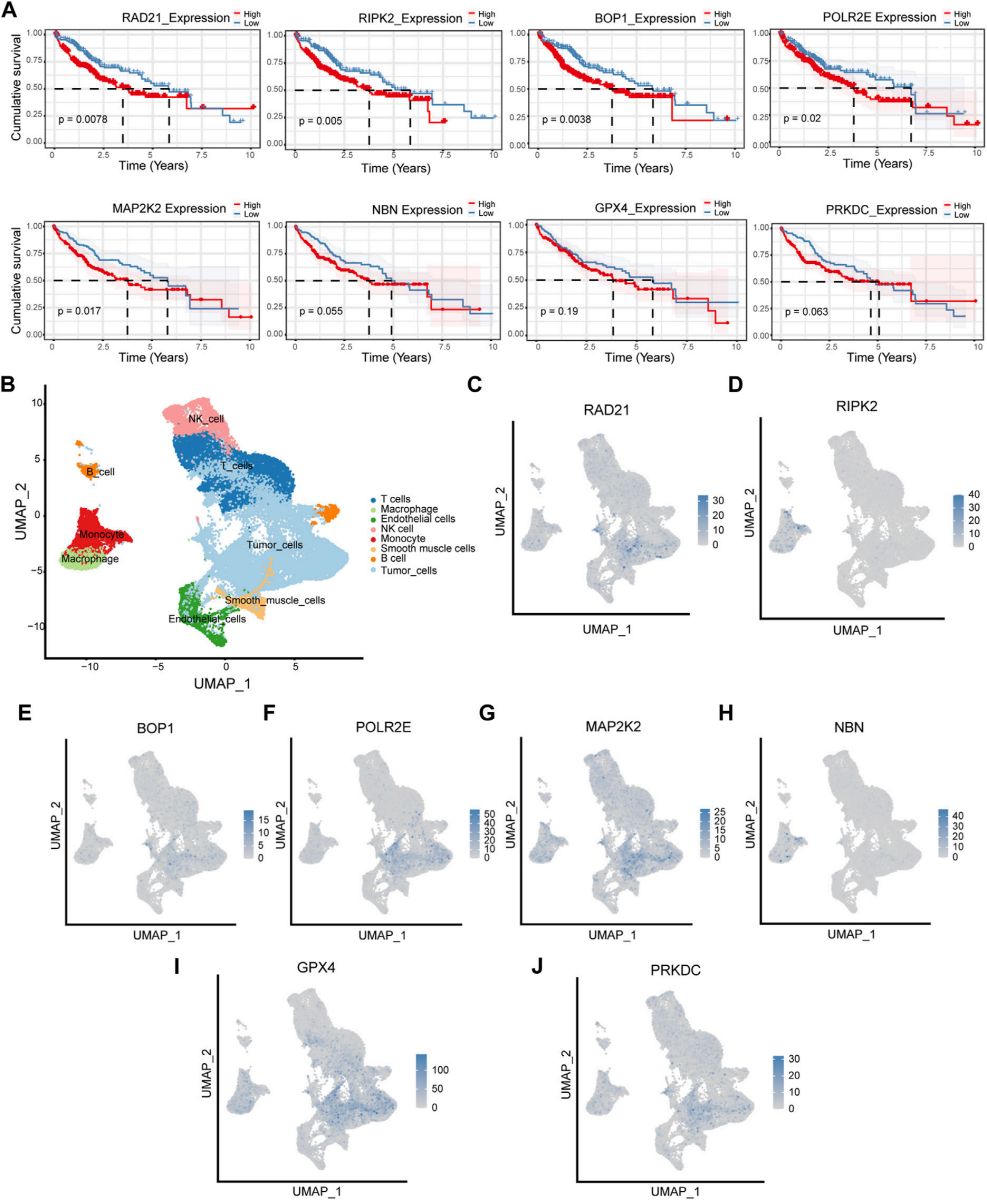
Survival analysis and cellular localization of the eight hub genes. **(A)** The survival analysis of eight hub genes in the TCGA cohort. **(B–J)** The expression of eight hub genes in different types of cells.

Survival analysis was performed for each of the eight hub genes. Compared with patients with low expression, those with high levels of RAD219 (*p* = 0.0078), RIPK2 (*p* = 0.005), BOP1 (*p* = 0.0038), POLR2E (*p* = 0.02), and MAP2K2 (*p* = 0.017) had significantly poorer outcomes (Figure 6A). To investigate the expression of the eight hub genes in various cell types, we conducted a single-cell sequencing analysis. As shown in Figures 6B–J, RAD21, BOP1, POLR2E, and PRKDC were mainly expressed in tumor cells, RIPK2 was mainly expressed in monocytes, MAP2K2 was mainly expressed in tumor cells and macrophages, NBN was mainly expressed in macrophages and monocytes, and GPX4 was mainly expressed in tumor cells and T cells.

### The NCPS scores are positively correlated with the levels of immune cell infiltration and the expression of immune checkpoint genes

As shown in the above analysis, patient outcomes varied significantly within the NCPS subgroups. To explore the reasons for this and inform immunotherapy, comparisons of the levels of immune cell infiltration between the various groups were conducted.

**FIGURE 7:**
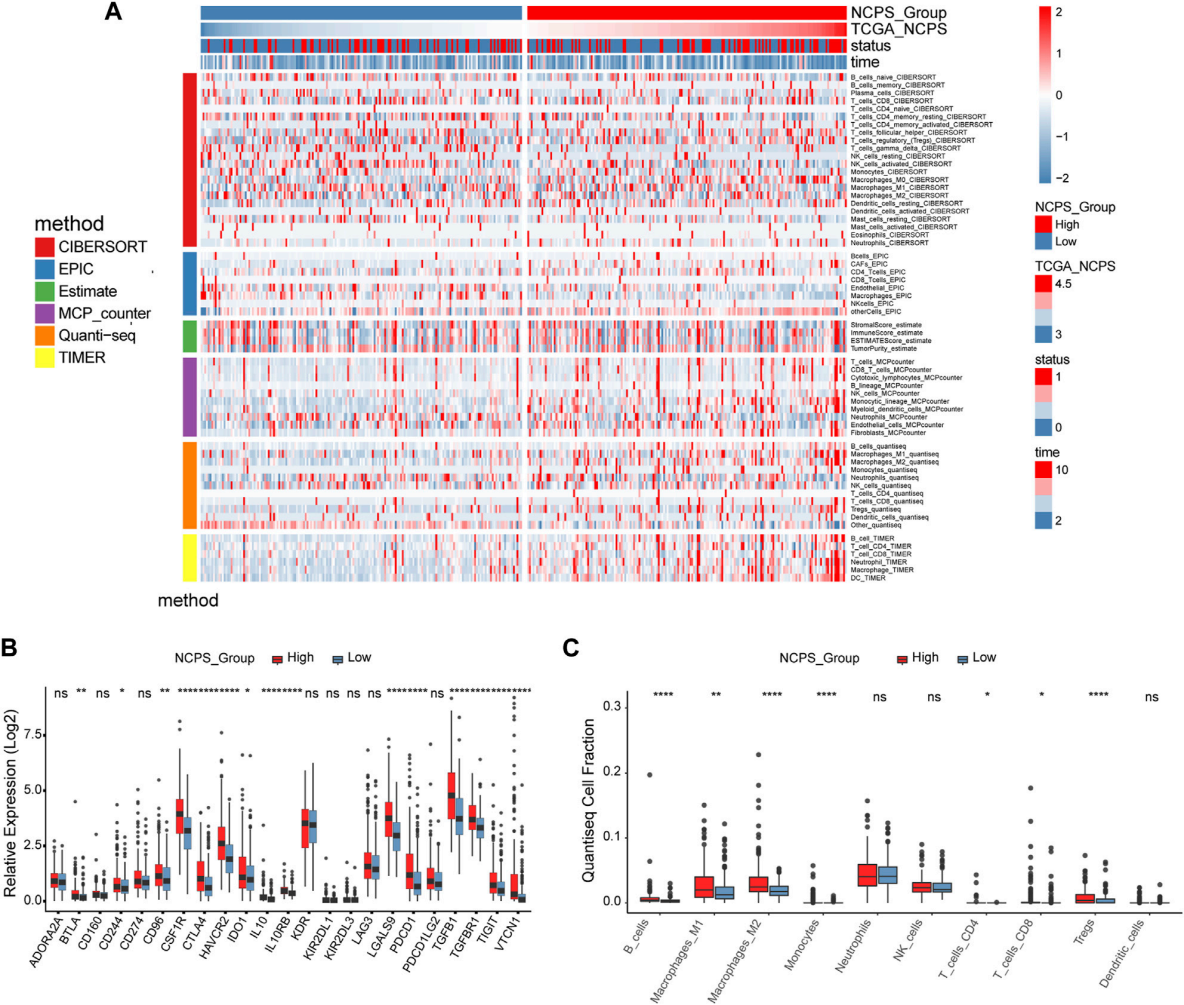
Immune infiltration analysis of TCGA cohort. **(A)** Heat map of immune cell infiltration in high and low NCPS groups with six immune infiltration algorithms. **(B)** Expression of immune checkpoint genes in high- and low-NCPS groups. **(C)** Results of the quanTIseq algorithm.

As shown in Figure 7A, six different immune infiltration algorithms have been used to estimate the relationship between necroptosis and immune cells. Specifically, the three algorithms of MCP counter, Quanti-seq, and TIMER clearly demonstrated that there were more immune cell infiltrations in the high-NCPS group, including macrophages, NK cells, T cells, monocytes, B cells, and dendritic cells. We then investigated the expression of genes associated with immune checkpoints. Figure 7B demonstrated that many immune checkpoint genes, such as PDCD1 and CTLA4, were more highly expressed in the high NCPS group. High NCPS patients were likely to have a higher degree of immune infiltration. However, patients with high-NCPS may suffer from low response states due to high levels of immune checkpoint genes, and immune checkpoint inhibitors may be of greater benefit to patients with such conditions. In addition, we examined the immune infiltration results obtained by different algorithms. The QuantTIseq algorithm showed that patients with high-NCPS levels had more macrophages, B cells, and T cells (Figure 7C).

### A high-NCPS score is associated with a greater incidence of gene mutations

**FIGURE 8:**
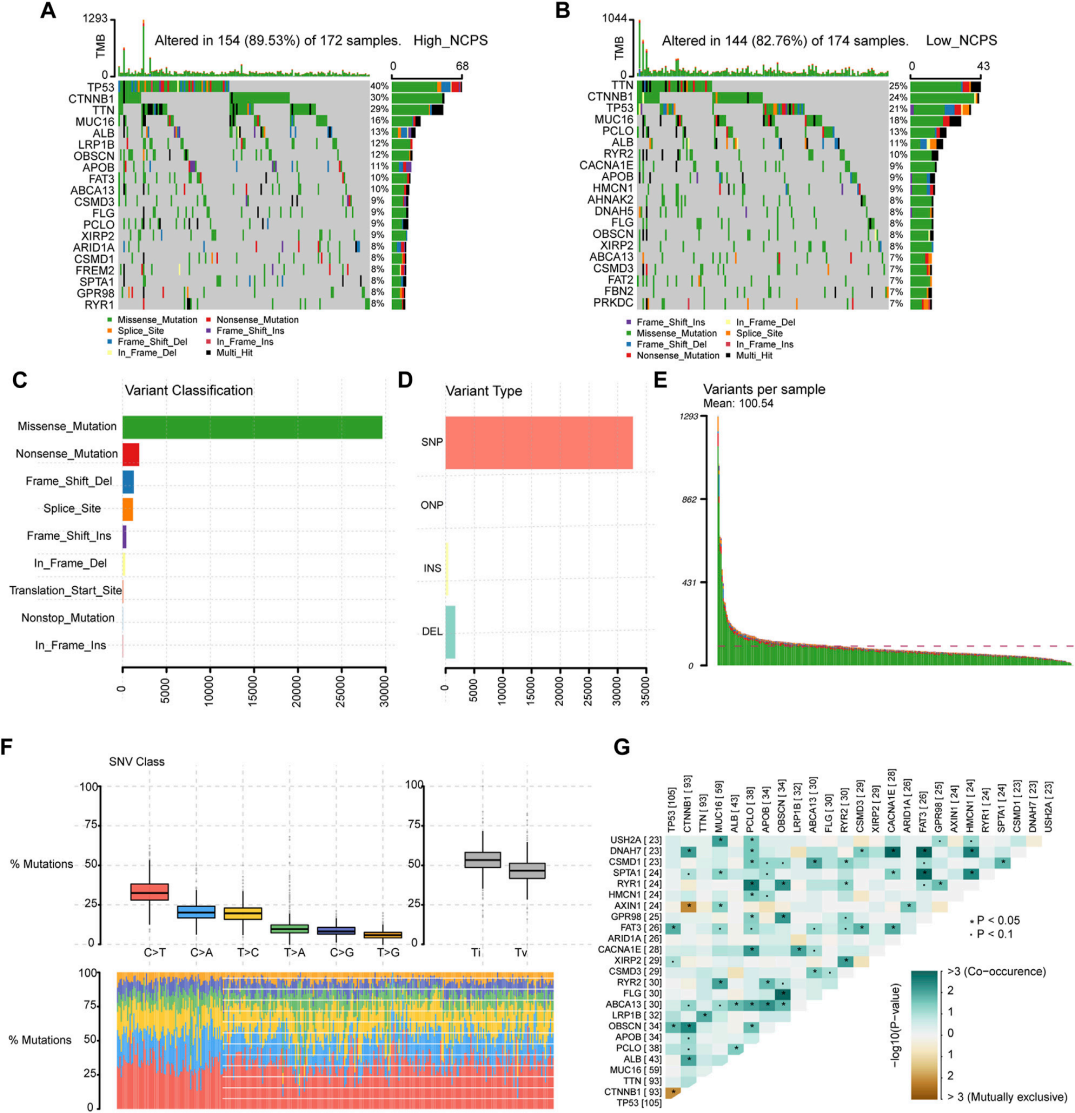
Mutation landscape of TCGA cohort. **(A,B)** Mutated genes in high- and low-NCPS groups. **(C,D)** Classification and types of variants. **(E)** Mutations in each sample. **(F)** The base mutation types of SNVs. **(G)** The correlation between pairs of mutated genes.

According to the NCPS scores in the high- and low-group, 20 of the top mutated genes were identified. As shown in Figures 8A,B, the incidence of mutations in the 20 most frequently mutated genes was 89.53% (High NCPS) and 82.76 % (Low NCPS) for the two groups. In the high-NCPS group, the highest mutation rates were PT53 (40%), CTNNB1 (30%), and TTN (29%). In the low NCPS group, TTN (25%), CTNNB1 (24%), and PT53 (21%) were the mutations with the highest rates. A higher incidence of mutations was observed in the high-NCPS group as compared to the low-NCPS group. Mutations were analyzed for eight hub genes (Supplementary Material S8). The highest Variant Classification shown in Figure 8C was Missense Mutation. Single nucleotide polymorphism (SNP) was the highest Variant Type (Figure 8D). Figure 8E indicated that an average of 100 genes were mutated in each sample. Figure 8F showed that the top three base mutation types of single nucleotide variants (SNVs) were C>T, C>A, and T>C. In addition, we analyzed the correlation between pairs of mutated genes. Figure 8G showed a strong co-relation between FLG and OBSCN (*p* < 0.0001, OR = 8.803), FAT3 and DNAH7 (*p* = 0.00064, OR = 6.925). There was a strong mutually exclusive relationship between CTNNB1 and TP53 (*p* = 0.00811, OR = 0.459), AXIN1 and CTNNB1 (*p* = 0.00733, OR = 0.109) (Supplementary Material S9).

### Drug sensitivity of HCC and hub gene protein expression are positively correlated with NCPS scores

**FIGURE 9:**
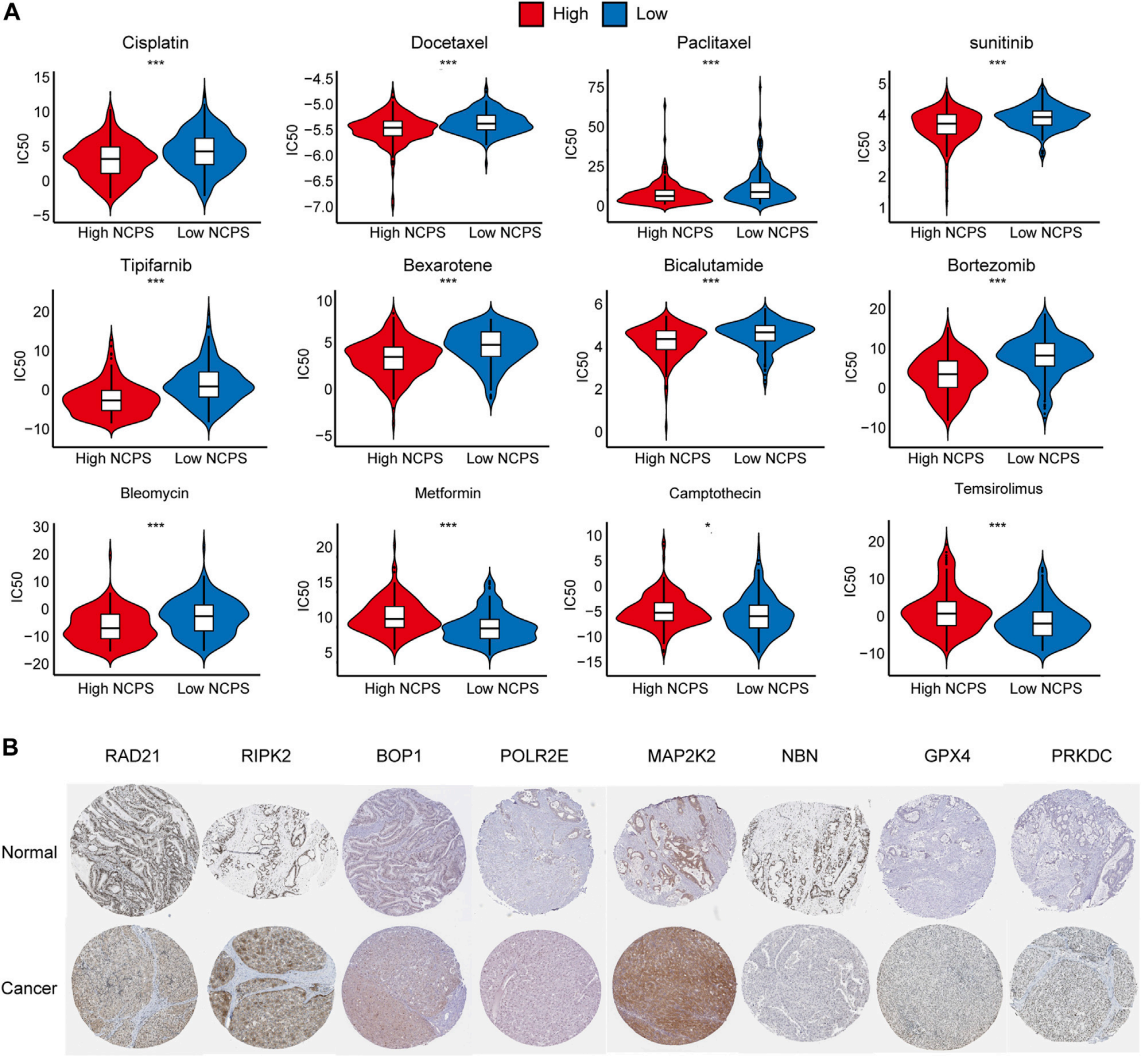
Drug sensitivity and Immunohistochemical analysis. **(A)** Drug sensitivity analysis in. high- and low-NCPS groups. **(B)** Immunohistochemical analysis of eight hub genes.

Based on the “pRRophetic” package, we assessed the sensitivity of different NCPS subgroups to drugs commonly used as a treatment for HCC. The high-risk group showed higher sensitivity to cisplatin, docetaxel, paclitaxel, sunitinib, tipifarnib, bexarotene, bicalutamide, bortezomib, and bleomycin, while the low-risk group showed higher sensitivity to metformin, camptothecin, temsirolimus (Figure 9A). The immunohistochemical analysis of the HPA database showed that protein products with high NCPS-related genes were expressed at higher levels in HCC samples compared to normal tissues (Figure 9B).

### Pathway enrichment and localization in single-cell sequencing data

**FIGURE 10:**
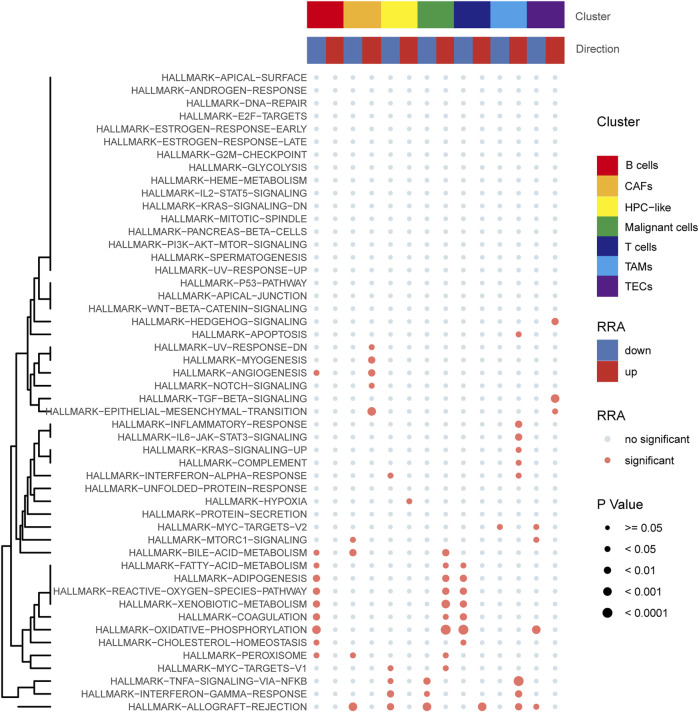
Pathway enrichment analysis of single-cell data.

**FIGURE 11:**
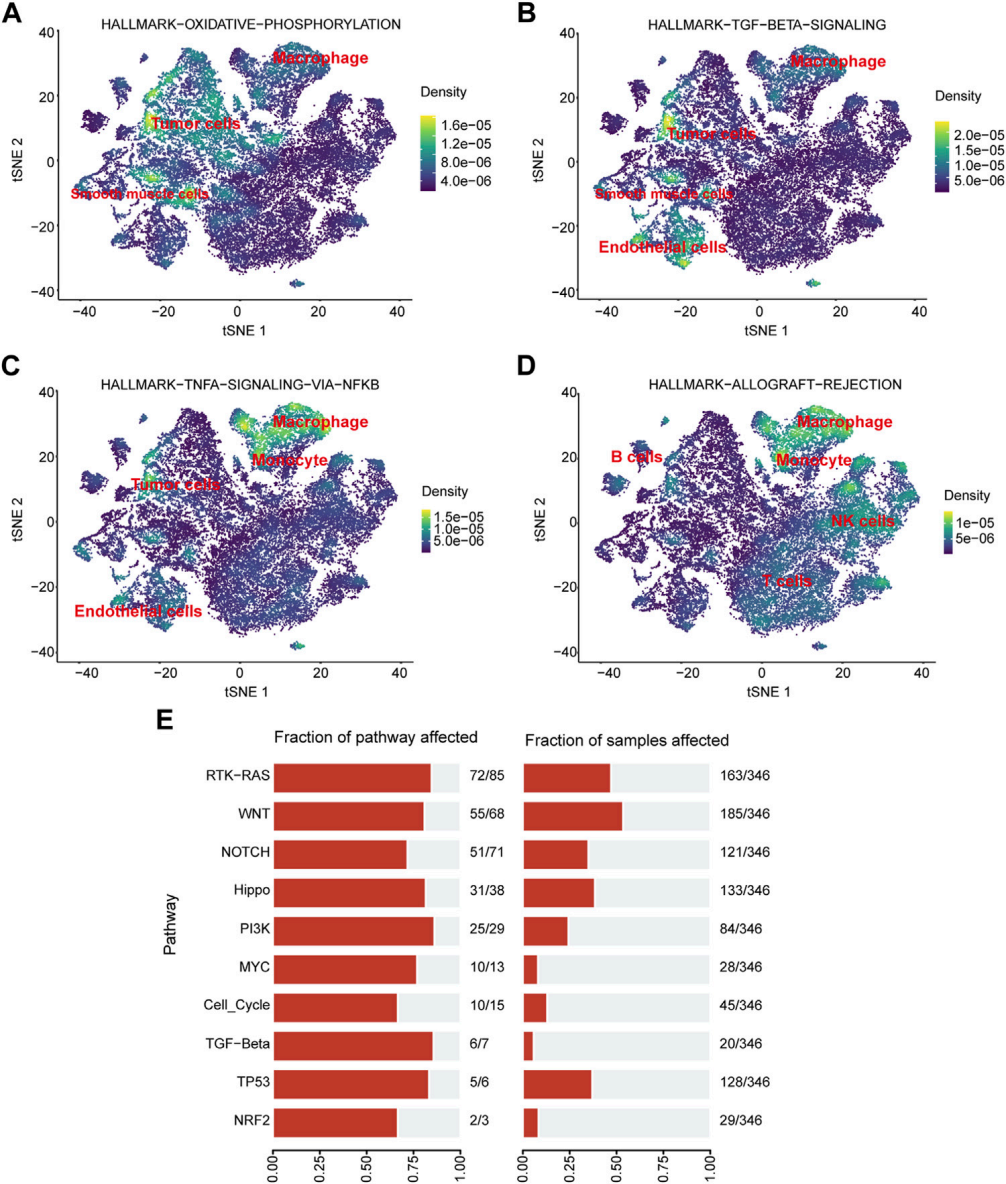
Pathway enrichment analysis. **(A–D)** Localization of different pathways in the single-cell dataset. **(E)** The number of pathways enriched in the TCGA cohort.

Pathway enrichment analysis of single-cell data revealed that HALLMARK OXIDATIVE PHOSPHORYLATION was upregulated in Malignant cells but downregulated in T cells, TECs, and B cells. HALLMARK ALLOGRAFT REJECTION was downregulated in Malignant cells, upregulated in T cells, downregulated in CAFs, and upregulated in TAMS. HALLMARK-TNFA -SIGNALING-VIA-NFKB was downregulated in Malignant cells and upregulated in TAMs. HALLMARK-TGF-BETA-SIGNALING was upregulated in TECs (Figure 10). In addition, we explored the expression of these signaling pathways in different cell types by single-cell sequencing analysis (Figures 11A–D) and profiled the pathways associated with disease (Figure 11E).

## Discussion

With increasing incidence, HCC has become the second leading cause of cancer-related deaths (Bray et al., 2018). Due to lifestyle changes, HCC has become the fastest growing cancer in developed countries, but the response to antitumor therapy is relatively poor. Approximately 50% of HCC patients receive systemic therapy, traditionally with first-line sorafenib or lenvatinib. In the past 5 years, immune checkpoint inhibitors have completely altered the treatment regimen for HCC and improved the prognosis (Llovet et al., 2022). The immune microenvironment plays a significant role in the progression of HCC, and HCC with high- and low-necroptosis respond differently to immune checkpoint inhibitor therapy. However, at present, there are no validated biomarkers to aid in clinical decision-making in this regard.

Immune checkpoint inhibitors are used because immune cells can receive inhibitory signals by activating immune checkpoint molecules. By activating immune checkpoint molecules to receive inhibitory signals, their activity and proliferation are blocked (Huang and Chang, 2019). These immune checkpoints can be used by cancer cells, leading to impaired immune surveillance (Liu and Qin, 2019). PD-1, PD-L1, and cytotoxic T cell antigen 4 (CTLA-4) are the main immune checkpoints that have been targeted by monoclonal antibodies.

Utilizing comprehensive data analysis on HCC datasets from TCGA, ICGC, and GEO databases, we built a prognostic profile for NCPS-related genes associated with HCC. We calculated risk scores to identify high- and low-risk groups of patients with HCC. All three cohorts both showed that the high-risk group did significantly worse than the low-risk group in HCC. Xie et al. (2022) found similar results in triple-negative breast cancer, indicating that the higher the NCPS score, the larger the tumor and the worse the prognosis. Furthermore, the ROC curve revealed that this feature might be accurate in predicting the prognosis of patients with HCC at 1, 3, and 5 years. Based on the immune microenvironment analysis, immunotherapy was more likely to be effective in necroptosis with higher expression levels. The low response to immunotherapy of HCC could be attributed in part to the low mutational load and the generation of new immune checkpoints (Ricciuti et al., 2019; Scheiner et al., 2022). Therefore, it becomes fascinating to explore the immune microenvironment of HCC. Necroptosis may play an important role in TME by the release of inflammatory molecules during the induction of apoptosis. However, it remains unclear whether necroptosis plays a role in HCC.

Necroptosis is a necrotic programmed cell death that is powerfully immunogenic and participates in a complex interplay of autophagy and apoptosis (Gong et al., 2017). There is growing evidence that necroptosis plays an important role in prognosis, disease progression and tumor metastasis, and immune surveillance in cancer patients (Gong et al., 2019). Targeting necroptosis through immune checkpoint is also emerging as a new approach in tumor therapy.

The role of necroptosis in cancer is complex. It is still unclear exactly what role necroptosis plays in cancer. In general, high expression of necroptosis elicits strong adaptive immune responses that can inhibit tumor progression (Yatim et al., 2015). However, these recruited strong immune responses may also promote tumor progression. The inflammatory response may promote tumorigenesis and metastasis, as well as may generate an immunosuppressive tumor microenvironment (Seifert and Miller, 2017). Therefore, it is essential to investigate the molecular mechanisms and physiopathological aspects of necroptosis, as well as its interaction with immunity. In addition, it is imperative to discover the correlation between specific necroptosis markers and the prognosis of HCC. This is to unravel the confusion of necroptosis correlation in HCC and further develop targeted antitumor therapeutic drugs. In this study, combining single-cell analysis and second-generation sequence analysis, we were able to identify a significant difference between NCPS groups in terms of immune cell infiltration in HCC. Significant differences were observed between the high- and low-NCPS groups. In addition, the study findings indicated that a high level of NCPS group corresponds to a high level of immune checkpoint gene expression. Therefore, patients with HCC who have a high NCPS are more likely to respond to immunotherapy.

The datasets GSE125449 and GSE151530 have been initially explored to reveal changes in the immune microenvironment of HCC. Among the published results, GSE125449 reveals different degrees of heterogeneity of malignant cells within and between tumors and different TME landscapes by single-cell sequencing techniques. GSE151530 provides insights into the collective behavior of HCC cell communities by single-cell sequencing and potential tumor evolution in response to therapy drivers. We first classified HCC cells into two groups based on their NCPS scores by analyzing single cells of GSE125449 and GSE151530. This provided a reference for us to study the heterogeneity of necroptosis in HCC. Based on these two cell populations, we calculated the differentially expressed genes, which then served as a basis for constructing a prognostic model. For the validation of the prognostic model, survival data from the ICGC dataset was analyzed.

Our study has some limitations. First, a comprehensive analysis of HCC tissues is needed to fully validate how the eight NCPS-related genes are involved in the development of HCC. This was not examined in the current study. Second, further validation with larger patient datasets is needed better to estimate the accuracy of the model’s predictions. Finally, further experimental evidence is needed to fully understand the role and mechanisms of eight NCPS-related genes in HCC.

## Conclusion

Through the analysis of single-cell and bulk multi-omics sequencing data, we constructed a prognostic model related to necroptosis and explored the relationship between high- and low-necroptosis groups and immune cell infiltration in HCC. This provides a new reference for further understanding the role of necroptosis in HCC. This may be useful in developing new therapeutic targets for the treatment of HCC. However, further molecular experiments are required to confirm the present findings.

## Data Availability

The original contributions presented in the study are included in the article/Supplementary Material, further inquiries can be directed to the corresponding author.
