# Supplementary material for: A necroptosis-related prognostic model for predicting prognosis, immune landscape, and drug sensitivity in hepatocellular carcinoma based on single-cell sequencing analysis and weighted co-expression network
Source: Front Genet. 2022 Sep 21;13:984297. doi: 10.3389/fgene.2022.984297 (PMC9533069; doi:10.3389/fgene.2022.984297)
Supplement: Supplementary file 2 [file DataSheet8.ZIP › Supplementary Material S8.pdf]

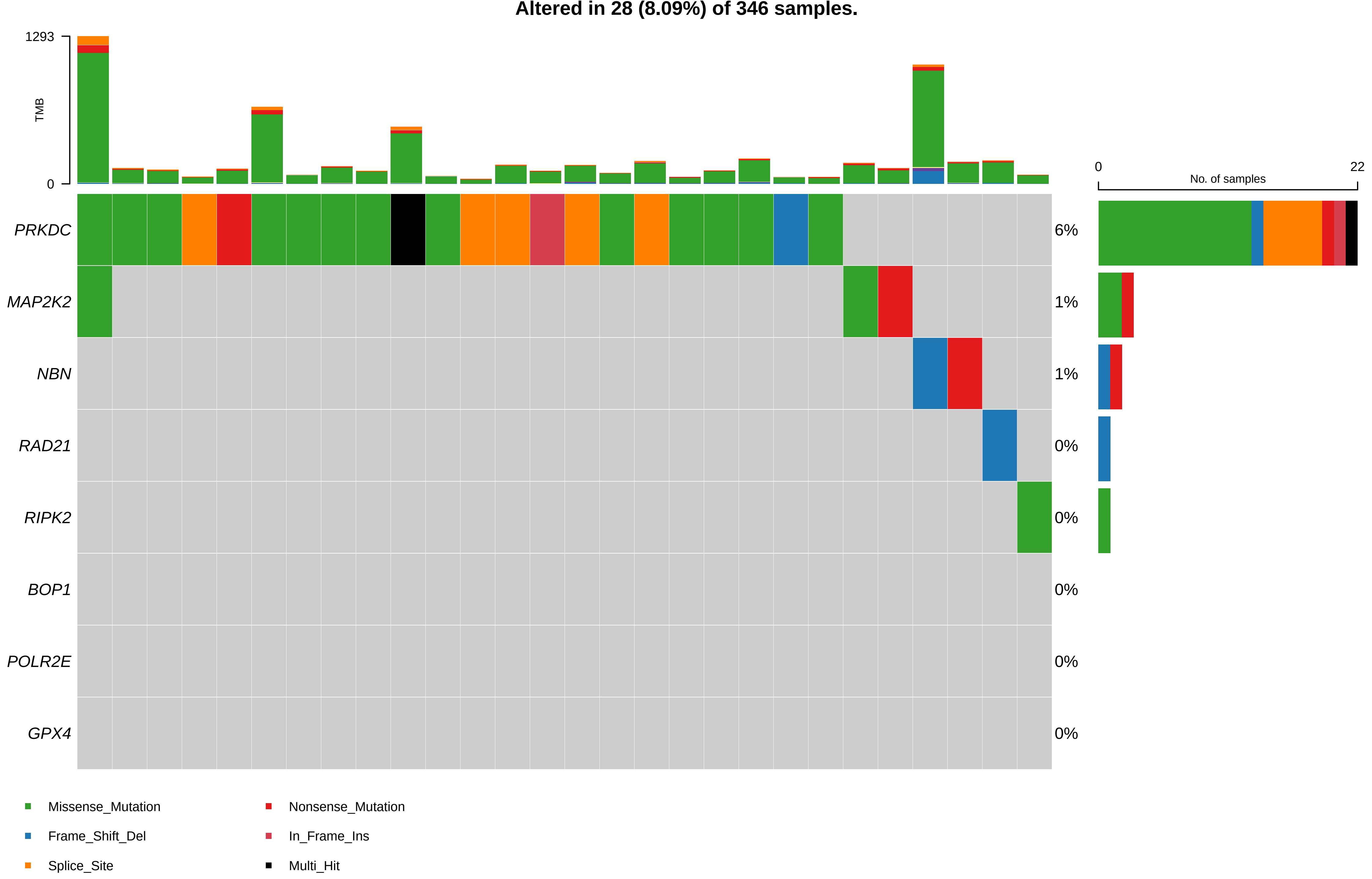
Altered in 28 (8.09%) of 346 samples.
1293
TMB
0
PRKDC
MAP2K2
NBN
RAD21
RIPK2
BOP1
POLR2E
GPX4
6%
1%
1%
0%
0%
0%
0%
0%
0
22
No. of samples
Missense_Mutation
Nonsense_Mutation
Frame_Shift_Del
In_Frame_Ins
Splice_Site
Multi_Hit
