# Supplementary material for: A necroptosis-related prognostic model for predicting prognosis, immune landscape, and drug sensitivity in hepatocellular carcinoma based on single-cell sequencing analysis and weighted co-expression network
Source: Front Genet. 2022 Sep 21;13:984297. doi: 10.3389/fgene.2022.984297 (PMC9533069; doi:10.3389/fgene.2022.984297)
Supplement: Supplementary file 5 [file DataSheet1.ZIP › Supplementary Material S1.docx]

**Methods**

- 1. **Download and processing of transcriptome data**

This flowchart illustrates the key steps in the analysis (**Supplementary Material S1**). The data of HCC were downloaded from TCGA (<https://portal.gdc.cancer.gov/>) as a training cohort(Grossman et al., 2016). Count data and TPM data of HCC were extracted using R software (4.2.0) (TPM is available in the new version of TCGA database). Then mRNA was extracted, and mean values were taken for the same genes A total of 363 tumor samples with complete clinical data were obtained(Zhang et al., 2019). When processing clinical data, samples with identical information were removed, HCC samples screened in the previous step were matched with clinical data and common samples were extracted, and mRNA expression matrices (TPM and counts data types) of the final samples were extracted for the next step of analysis.

The HCC dataset was downloaded through ICGC(<https://dcc.icgc.org/>) database as a validation cohort. Count data of mRNA was extracted, and the mean value was taken for the same genes. Next, the mRNA sequencing data of the ICGC cohort was converted into transcripts per kilobase million (TPM) values, and then log2(x + 1) transformed, which was suggested to be the most accurate quantification method with minimal statistical biases. A total of 240 tumor samples were obtained with complete clinical information. When processing the clinical data, samples with identical information were removed. HCC samples screened in the previous step were matched with the clinical data and the common samples were extracted, and the mRNA expression matrix of the final samples was extracted for the next step of analysis.

GSE76427, measured using the Illumina HumanHT-12 V4.0 expression beadchip, contained 115 HCC samples(Grinchuk et al., 2018). The raw CEL files for GSE76427 were downloaded from the GEO database(<https://www.ncbi.nlm.nih.gov/geo/>). The R packages, “affy” and “lumi,” were used to calculate background-adjusted, quantile-normalized, and probe- level data-summarized values for all probe sets(Irizarry et al., 2003; Du et al., 2008). The “ComBat” algorithm in the “sva” package was used to correct batch effects from nonbiological technical biases(Leek et al., 2012). Based on the GPL10558 platform, gene symbols were annotated to the Illumina probe ID from the microarray data. The mean expression value was considered for probes that mapped to a single gene.

- 1. **Download and processing of single-cell data**

The single-cell datasets GSE125449 and GSE151530 for HCC were downloaded from the GEO database(Barrett et al., 2013). The GSE125449 dataset contains 9 HCC samples and the GSE151530 dataset contains 32 HCC samples. We performed quality control on the data of all samples. We retained cells with genes expressed in at least 10 cells, less than 10% of mitochondrial genes, more than 200 genes, less than 5% hemoglobin genes, less than 50% ribosomal genes, and expression between 200 and 7000. We set a limit of 3000 highly variable genes. Next, we normalized all samples, removed batch effects, and integrated them by SCT. Then, using the tSNE method with the "DIMS" parameter set to 20, the dimensionality of the data was reduced. Cell clustering was then carried out using the "KNN" method with a resolution of 2.0. Subsequently, the cells were annotated with the Human Primary Cell Atlas (HPCA) from the "SingleR" package as a reference dataset(Mabbott et al., 2013). Finally, the proportion of NCPS-related genes in each cell can be calculated using the "PercateFeatureSet" function.

**Reference**

Barrett, T., Wilhite, S.E., Ledoux, P., Evangelista, C., Kim, I.F., Tomashevsky, M., et al. (2013). NCBI GEO: archive for functional genomics data sets--update. *Nucleic Acids Research* 41(Database issue)**,** D991-D995. doi: 10.1093/nar/gks1193.

Du, P., Kibbe, W.A., and Lin, S.M. (2008). lumi: a pipeline for processing Illumina microarray. *Bioinformatics (Oxford, England)* 24(13)**,** 1547-1548. doi: 10.1093/bioinformatics/btn224.

Grinchuk, O.V., Yenamandra, S.P., Iyer, R., Singh, M., Lee, H.K., Lim, K.H., et al. (2018). Tumor-adjacent tissue co-expression profile analysis reveals pro-oncogenic ribosomal gene signature for prognosis of resectable hepatocellular carcinoma. *Molecular Oncology* 12(1). doi: 10.1002/1878-0261.12153.

Grossman, R.L., Heath, A.P., Ferretti, V., Varmus, H.E., Lowy, D.R., Kibbe, W.A., et al. (2016). Toward a Shared Vision for Cancer Genomic Data. *The New England Journal of Medicine* 375(12)**,** 1109-1112. doi: 10.1056/NEJMp1607591.

Irizarry, R.A., Bolstad, B.M., Collin, F., Cope, L.M., Hobbs, B., and Speed, T.P. (2003). Summaries of Affymetrix GeneChip probe level data. *Nucleic Acids Research* 31(4)**,** e15.

Leek, J.T., Johnson, W.E., Parker, H.S., Jaffe, A.E., and Storey, J.D. (2012). The sva package for removing batch effects and other unwanted variation in high-throughput experiments. *Bioinformatics (Oxford, England)* 28(6)**,** 882-883. doi: 10.1093/bioinformatics/bts034.

Mabbott, N.A., Baillie, J.K., Brown, H., Freeman, T.C., and Hume, D.A. (2013). An expression atlas of human primary cells: inference of gene function from coexpression networks. *BMC Genomics* 14**,** 632. doi: 10.1186/1471-2164-14-632.

Zhang, J., Bajari, R., Andric, D., Gerthoffert, F., Lepsa, A., Nahal-Bose, H., et al. (2019). The International Cancer Genome Consortium Data Portal. *Nature Biotechnology* 37(4)**,** 367-369. doi: 10.1038/s41587-019-0055-9.
