# Supplementary figures and images for: A necroptosis-related prognostic model for predicting prognosis, immune landscape, and drug sensitivity in hepatocellular carcinoma based on single-cell sequencing analysis and weighted co-expression network
Source: Front Genet. 2022 Sep 21;13:984297. doi: 10.3389/fgene.2022.984297 (PMC9533069; doi:10.3389/fgene.2022.984297)

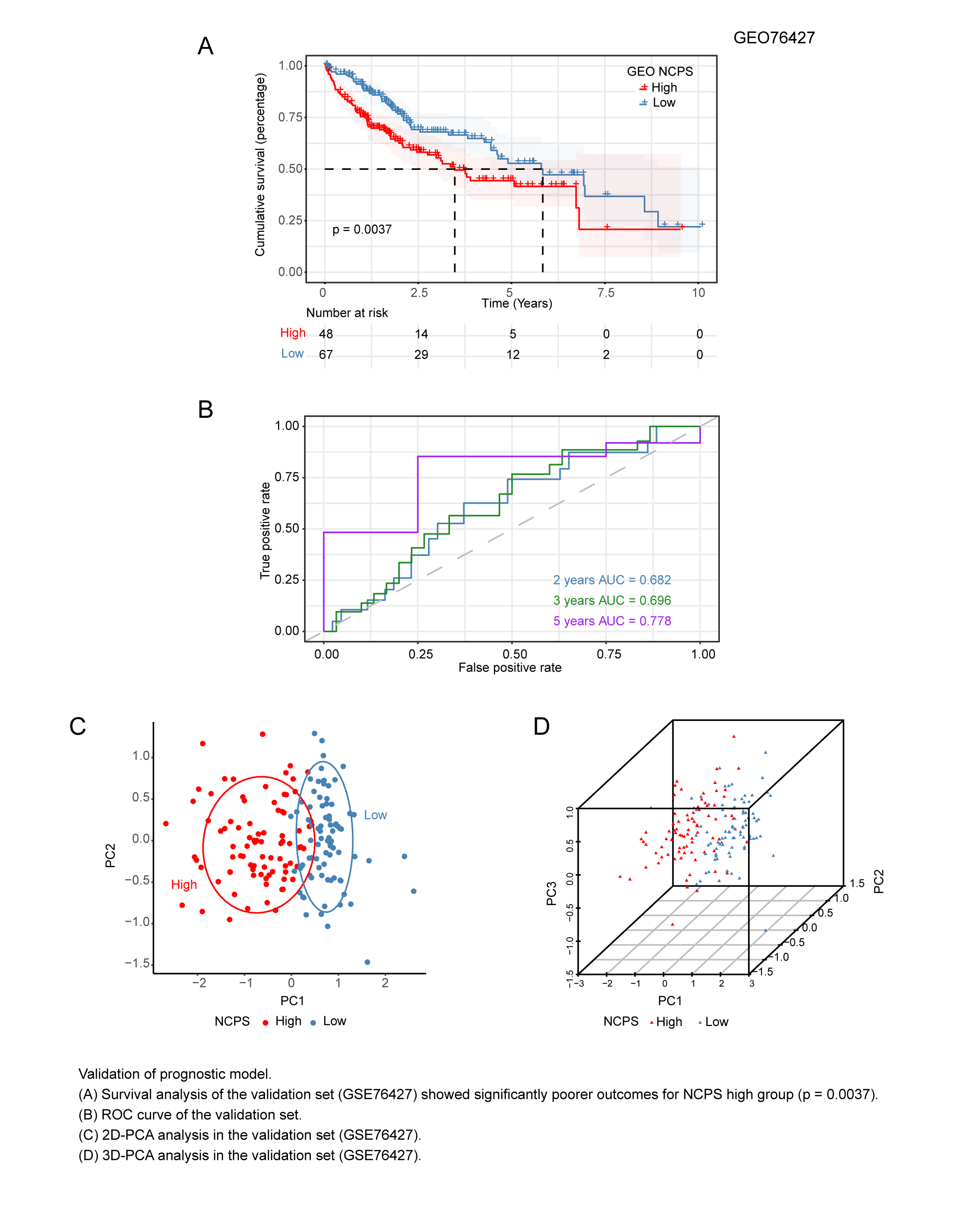

Supplement: Supplementary file 7 [file DataSheet6.ZIP › Supplementary Material S6.tif]
